# Direct PCR: a new pharmacogenetic approach for the inexpensive testing of HLA-B*57:01

**DOI:** 10.1038/tpj.2014.48

**Published:** 2014-09-09

**Authors:** R Cascella, C Strafella, M Ragazzo, S Zampatti, P Borgiani, S Gambardella, A Pirazzoli, G Novelli, E Giardina

**Affiliations:** 1 Department of Biomedicine and Prevention, School of Medicine, University of Rome ‘Tor Vergata', Rome, Italy; 2 Neuromed IRCCS, Pozzilli, Italy; 3 ViiV Healthcare, Verona, Italy; 4 Molecular Genetics Laboratory UILDM, Santa Lucia Foundation, Rome, Italy

## Abstract

One of the most successful applications of pharmacogenetics research is the genetic screening for HLA-B*57:01, strongly associated with an increased risk to develop hypersensitivity reaction in HIV-positive patients following abacavir administration. Taking into consideration the limits of current genotyping methodologies, we have developed and validated (150 buccal swabs) an inexpensive pharmacogenetic approach for HLA-B*57:01 typing. In our assay DNA extraction and amplification are combined in one single step (direct PCR protocol), which is performed directly on the biological sample without the need of extraction and sequencing passages. The amplicons obtained by direct PCR can be easily separated on the agarose gel under ultraviolet. As per our results, the direct PCR represents a good alternative to the traditional methods of HLA-B*57:01 pharmacogenetic test, especially for those laboratories or countries where currently available approaches are often not available or not affordable. Furthermore it is an innovative approach, promoting a personalized, safer and cost-effective therapy.

## Introduction

In recent years the knowledge of the wide human genome variability has been even connected to the different individual drug response. Several studies described how genetic variants are involved in the regulation of sensitivity and safety of pharmaceutical treatments. Some of the most investigated genetic variants controlling the drug metabolism and toxicity are localized right on *CYP2D6* gene, coding for the CYP2D6 enzyme. It catalyzes the metabolism of a large number of clinically important drugs, including antidepressants, neuroleptics, some antiarrhythmics, lipophilic β-adrenoceptor blockers and opioids. However, genetic polymorphisms can also modify the drug response, increasing the risk for developing adverse drug reactions, such as liver toxicity or hypersensitivity.^1^ Thus, the discovery of several genetic variants modulating the drug molecular pathways and the final effectiveness highlights the importance of interindividual variability. On the basis of these information, a new healthcare concept, referred as personalized medicine, has been conceived. The personalized medicine is characterized by a proactive and preventive approach, in contrast with the common reactive healthcare model.^2^ The new concept substantially aims to the quantification of wellness and the disease demystification, promoting the maintenance of the welfare state. It relies on the disease prevention in healthy people as well as on the improvement of patients' quality of life.^2^ The innovative healthcare model requires an integrative and dynamic medical analysis of the patient conditions, taking into consideration not only his clinical picture but also his genetic makeup.^1, 2, 3, 4, 5, 6^ Hence, the discovered genomic signatures may be employed as predictive, diagnostic and pharmacogenetic biomarkers.

Recently, many pharmacogenomic biomarkers were identified and translated into clinical practice, affecting the usage of drugs such as carbamazepine, warfarin and abacavir (ABC) via drug label updates.

The successful implementation of the pharmacogenomic biomarkers requires the careful design of specific and sensitive molecular assay. However, a gap still exists between the scientific knowledge generated and the clinical application. It may be explained by the fact that the successful incorporation of a pharmacogenetic test into routine practice requires a combination of high-level scientific evidence with widespread availability of reliable, easy to use cost-effective laboratory tests.

One of the best qualified pharmacogenomic marker is HLA-B*57:01 allele to predict hypersensitivity reaction (HSR) to ABC.^7,8^ ABC is an antiretroviral drug, highly effective in the treatment of HIV infection. Precisely it belongs to the nucleoside reverse transcriptase inhibitors class of antiretroviral drugs, preventing from viral DNA replication and thereby it reduces the viral load. ABC presents a good oral bioavailability (83%), allowing it to be easily administered by tablets. ABC effectiveness has been demonstrated to be higher in combination with other antiretroviral drugs, such as lamivudine and zidovudine.^9^ Despite its elevated efficiency, ABC is not usually preferred due to the risk for developing HSR. This adverse reaction affects about 5% of treated patients within the first 6 weeks of treatment. It is a multiorgan syndrome characterized by gastrointestinal and respiratory symptoms, fever, rush and even death in case of ABC rechallenge.^10^ A very extensive research have documented the strong association between HSR to ABC in relation to the HLA-B*57:01 allele.^8,11, 12, 13^ This correlation led to look at HLA-B*57:01 as a potential pharmacogenomic biomarker to be screened before ABC administration, with the purpose of reducing ABC hypersensitivity.^4,6,13,14^ On this subject, retrospective and perspective studies confirmed that the HLA-B*57:01 genotyping prior to drug administration decreases the incidence of HSR in patients.^11,12^ Nevertheless it is important to remark that HLA-B*57:01 frequency is different among populations. The higher prevalence of the allele at risk has been registered in Indian ethnic groups (4–10%), while the Chinese, Korean and Japanese populations present the minor frequency (0–0.3%). The Caucasian group has a HLA-B*57:01 prevalence of 5–8% whereas the 1–2% has been estimated for African and Afro-American ethnic groups.^15^

Although HLA-B*57:01 frequency variability, the screening is still useful even in areas presenting low frequency, to avoid HSR diagnosis wrongly associated to ABC treatment.^11,12,14,16^ These data highlighted thereby the clinical validity and utility of HLA-B*57:01 screening into routine analytical practice.^11, 12, 13, 14,16^ This wealth of data supported in 2008 change in Summary of Product Characteristics, European Medicines Agency of ABC containing products to introduce a mandatory screening for HLA-B*57:01 prior to drug administration.

The genetic screening for HLA-B*57:01 represents an excellent example of successful translation of pharmacogenetic research into the clinical practice, encouraging pharmacogenetic test adoption and development. The progressive implementation of the screening has paralleled with the development of innovative methods for HLA-B*57:01 identification, each one with pros and cons.^17^

Although the clinical usefulness of the pharmacogenetic test, its diffusion in the less industrialized countries is limited, because of the high costs required for genotyping and instrumental equipment. In order to promote the availability of this test even in less industrialized countries, here we show a new protocol for HLA-B*57:01 test enabling the genotyping at <1 euro each.

## Materials and methods

In our assay DNA extraction and amplification (direct PCR protocol) are combined in one single step, reducing the time, cost and the rate of human errors. The system is also designed to be performed with basic and economic instrumental equipment.

The direct PCR protocol (Thermo Scientific Phusion Human Specimen Direct PCR kit, Finnzymes, Finland) consists of a unique phase of DNA extraction/amplification, directly from the biological sample, without the need for expensive and time consuming extraction and sequencing procedures. The enzyme selected for the PCR step is the Phusion Hot Start II high-fidelity DNA polymerase' (Finnzymes, Finland). The high resistance to the inhibitors commonly found in the biological samples and the elevated enzymatic activity, make this polymerase robust, rapid and accurate. The amplicons obtained by PCR can be easily separated by electrophoresis and visualized on agarose gel, under ultraviolet. The protocol allows using of DNA sources less infective (in case of HIV presence) than blood such as saliva or buccal swab.

Taking into account the previous experience in the study of HLA-B*57 region and in the diagnostic practice of our laboratory, we chose oligonucleotides for the specific amplification of HLA-B*57 region (namely 1F: 5′-GTCTCACATCATCCAGGT-3′ and R-seq: 5′-CGTCTCCTTCCCGTTCTC-3′, Applied Biosystems, Warrington, UK) and its subtype HLA-B*57:01 (referred as 1F: 5′-GTCTCACATCATCCAGGT-3′, 2R: 5′-ATCCTTGCCGTCGTAGGCGG-3′ and 3R: 5′-ATCCTTGCCGTCGTAGGCAG-3′, Applied Biosystems, Warrington, UK).^18^ As internal control, two primers (INT-F: 5′-GCAGAAGGGCGCATTATAGC-3′ and INT-R: 5′-CATGTGAGTACACTCAGCTTG-3′, Applied Biosystems, Warrington, UK) were designed for the amplification of the *LOXL-1* gene.

Considering the variability of the frequency of HLA-B*57:01 in populations with low and middle incomes we decided to use the direct PCR and gel electrophoresis as the first-line protocol to detect the positivity/negativity for HLA-B*57. After a number of experiments we found the optimal concentrations for primers 1F and R-seq as well as the best PCR cycle. The amplicons of *LOXL-1* and HLA-B*57, 444 and 262 bp in size respectively, allow to detect the positivity/negativity for HLA-B*57 locus on 2.5% agarose gel (Lonza, Rockland, ME, USA). Saliva and buccal swabs taken from subjects with known genotype were utilized as DNA sources. The PCR mix was prepared in a total volume of 20 μl, using the PCR reagents (the buffer containing MgCl_2_ and deoxynucleotide triphosphates and the polymerase) provided with the Thermo Scientific Phusion Human Specimen Direct PCR kit. The optimal concentration of primers was 8 pmol μl^−1^ for 1F-R-seq and 2 pmol μl^−1^ for INT-F–INT-R. The PCR cycle was fixed at 98° for 5 min; 98° for 10 s, 60° for 30 s and 72° for 30 s. The last three steps were repeated 32 times with a final elongation step at 72° for 10 min.

For the specific detection of HLA-B*57:01 allele, a nested PCR has been projected.

The amplicons derived by direct PCR were used as templates for the second PCR, including specific primers (1F-2R-3R) for HLA-B*57:01 amplification. The ‘Taq Gold PCR' reagents (including the buffer, MgCl_2_, deoxynucleotide triphosphates and the Taq Gold; Applied Biosystems, Warrington, UK) were used for this second amplification step. Two reverse primers and one forward were selected to increase the accuracy of the genotyping analysis. In fact, the sequences of 2R and 3R differ only for a single base in 3′ position and code for HLA-B*57:01:01 and HLA-B*57:01:02, respectively. The final volume of the nested PCR was 16 μl.

A number of experiments were conducted in order to establish a specific and sensitive nested-PCR protocol. From these trials the optimal concentrations of primers resulted to be 0.2 pmol μl^−1^ for 1F and 0.15 pmol μl^−1^ for 2R and 3R, respectively. The PCR cycle was fixed as follows: 95° for 5 min, 94° for 30 s, 60° for 1 min, 72° for 1 min (the last three steps were repeated for 30 cycles) and 72° for 10 min. Even in this case the nested-PCR products can be verified on 2.5% agarose gel under ultraviolet after separation by electrophoresis (120 V for ~40 min). However in this case, over the bands corresponding to *LOXL-*1 and HLA-B*57, it could be observed a third band, equivalent to the amplification of HLA-B*57:01 (94 bp).

Successively the specificity of the nested PCR was evaluated, by sequencing of the HLA-B*57-positive subjects. The DNA for sequencing analysis was obtained through the extraction of DNA from the agarose gel, using the NucleoSpin Gel and PCR Clean-up (Macherey-Nagel, Duren, Germany).

PCR sequence reactions were performed with BigDye Terminator v3.1 (Applied Biosystems, Warrington, UK) according to the manufacturer's instructions. After purification with BigDye XTerminator (Applied Biosystems, Warrington, UK), samples were run on an ABI 3130xl (Applied Biosystems, Warrington, UK).

The specificity and the sensitivity of direct PCR was improved by testing the ‘dilution' version suggested by the manufacturer. It consists of an additional, brief pretreatment of the biological sample with a solution composed of 50 μl dilution buffer, 1.5 μl DNA release (both were provided with the reagents for direct PCR, Finnzymes, Finland) and 250 μl Tris-EDTA buffer at pH 8 (Promega, Madison, WI, USA). The samples were incubated with the solution at 98 °C for 2 min. At the end of the incubation, 1 μl of the pre-treated biological material is sufficient to perform the direct PCR.

## Results

**Figure 1:**
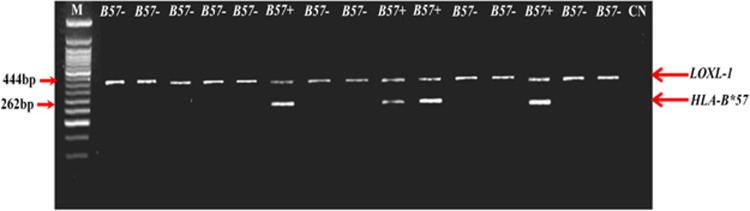
Direct PCR. M: ladder 50 bp. CN: negative control.

The direct-PCR trials proved to be able to discriminate HLA-B*57-negative subjects, representing >90% of the individuals, from the remaining ~7% of HLA-B*57-positive individuals. Figure 1 illustrates 15 samples analyzed by direct PCR and separated on the agarose gel. In particular the patients, non-HLA-B*57 (*n*=11, referred in the picture as ‘*B57−*') present the only amplification of the control gene (*LOXL-1*, 433 bp). The remaining samples (*n*=4, namely ‘*B57+*' in the picture) instead, clearly show amplicons corresponding to HLA-B*57 (262 bp) and *LOXL-1*.

Samples positive for HLA-B*57 were further typed for the presence of HLA-B*57:01 sequence by nested PCR. Taking into consideration the elevated variability of HLA-B*57 locus and the need of specific HLA-B*57:01 typing, two reverse primers were selected (2R-3R). Both primer sequences encode for HLA-B*57:01:01 and HLA-B*57:01:02 subtypes, respectively, ensuring more reliable results. In fact, HLA-B*57:01 really positive samples (independently from the variability of HLA-B*57:01 subtype) will find enough substrate to anneal to the template and proceed with the amplification. On the other hand, in HLA-B*57:01-negative subjects (for example, HLA-B*57:02, HLA-B*57:03 and HLA-B*58:01), the annealing of primers will be hampered by the lack of the right substrate producing thereby no detectable amplification on agarose gel.

**Figure 2:**
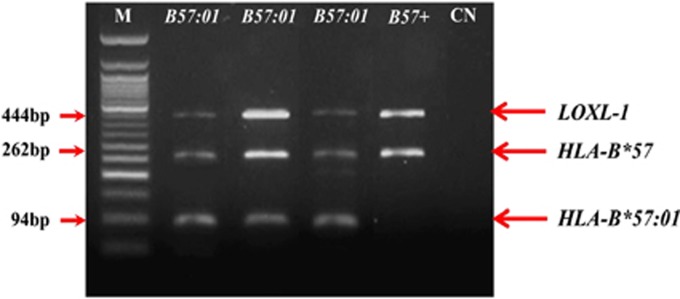
Nested PCR. M: ladder 50 bp. CN, negative control.

Figure 2 reports the results of the nested PCR conducted on the four HLA-B*57-positive samples previously found by direct PCR. In this case, the specific amplified regions allow us to distinguish the first three subjects as HLA-B*57:01-positive (94 bp, in the figure ‘*B57:01*'). The fourth one (in the figure ‘*B57+*') did not present any amplification of HLA-B*57:01 allele, although it showed to be HLA-B*57-positive.

**Figure 3:**
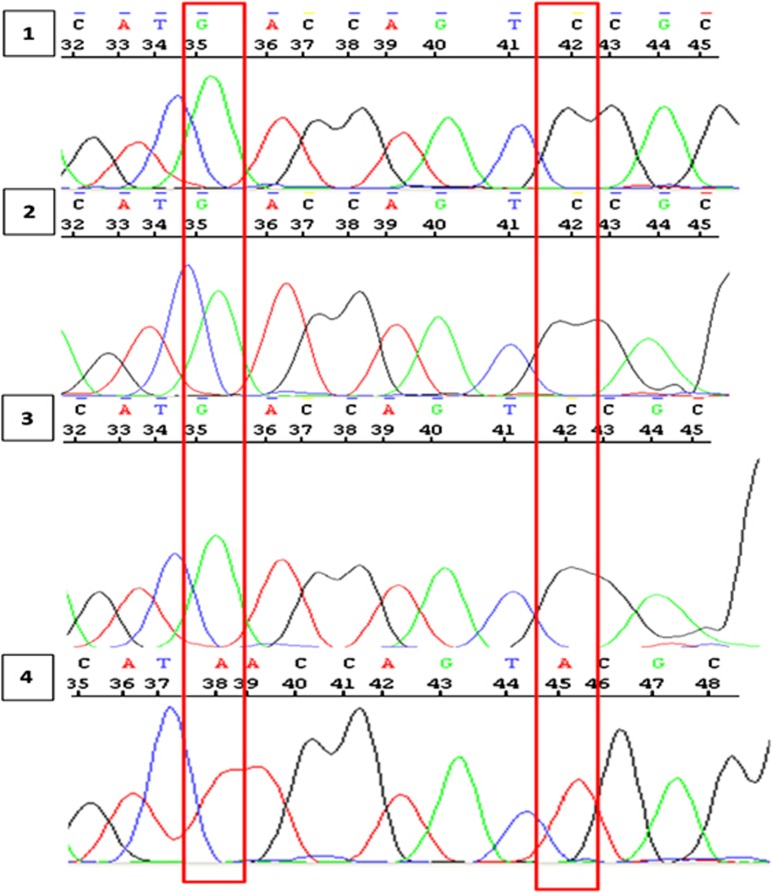
Electropherograms reporting the sequences of the nested-PCR products. The two bases distinguishing HLA-B*57:01 and HLA-B*57:02 alleles are highlighted in red.

Given these results, the samples processed by direct/nested PCR were subsequently sequenced after DNA extraction from the agarose gel in order to establish the correct genotype of the patients and thereby the accuracy of our molecular approach. As reported in Figure 3 and Table 1, the sequences of the first three subjects demonstrated to really correspond to the HLA-B*57:01 region, while the fourth one appeared to be HLA-B*57:02. These data were consistent with the genotypes identified by nested PCR and confirmed its ability to discriminate really positive HLA-B*57:01 subjects from individuals carrying different alleles; even those belonging to HLA-B*57 subtype, but not associated with the risk for ABC HSR.

Subsequently the ‘dilution protocol' has been chosen in order to optimize the output of the direct PCR. The treatment demonstrated to make the DNA more accessible to PCR and increase the final yield of the amplification reaction. The results obtained by numerous experiments showed that the dilution-direct approach is reproducible and standardized.

We next performed the validation of this test through the blind analysis of 150 buccal swabs. In particular, the samples came from 97 healthy patients and 53 HIV-positive subjects, although in this phase the genotype of the subjects was actually more important than the presence/absence of HIV infection. The cohort of patients (*n*=150) provided for the validation process was selected favoring the HLA-B*57:01-genotype despite the real frequency of the risk allele. The swabs were processed, in parallel, both by dilution-direct/nested PCR and the gold standard methodology (direct sequencing). Finally 68 patients were identified as HLA-B*57:01-positive. The concordance obtained by the comparison of the results derived from the two different methods (traditional sequencing vs dilution-direct PCR) was 100%.

After the development and the validation of the dilution-direct/nested-PCR assay, a number of experimental trials were performed to establish the optimal DNA source between saliva and buccal swab. Saliva presented a short half-life, strongly depending on its storage conditions and the sampling mode. The buccal swab, instead, showed to be more resistant and efficient, representing thereby the optimal DNA source for this pharmacogenetic test. Furthermore the sampling method is more practical, rapid and safer for the patient subjected to sampling as well as the operator handling these biological samples.

Taken altogether, these results provide evidence of the direct PCR as an inexpensive and efficient approach just requiring a PCR thermocycler and an electrophoretic chamber to perform the entire genotyping analysis.

## Discussion

ABC is one of the most effective antiretroviral drugs for the treatment of HIV, as it is available in the form of tablets (300 mg), it presents a long-term favorable toxicity profile as well as synergistic and additive effects when it is combined with other antiretroviral drugs (efavirenz and lamivudine).^14,19^ However its prescription is restricted because of the risk of HSR development after ABC ingestion in 5% of patients.^20^ As a matter of fact ABC is considered as a second-line drug in the treatment of HIV infection by the WHO (World Health Organization), particularly in pediatric patients.^21^ Although it is more utilized in developed countries, ABC is rarely employed in low- and middle-income regions, where HLA-B*57:01 frequency is even lower (for instance only 0.26% of the sub-Saharan African population carries the HLA-B*57:01 allele).^22,23^ In this case the genetic screening is still useful to exclude wrong HSR diagnosis that may discourage ABC prescription to patients who may benefit from this treatment. In this context the availability of an inexpensive, sensitive, pharmacogenetic predictive test for HSR prevention may represent a good strategy to improve the use of a highly efficient drug like ABC. To this purpose the development of our direct PCR represents a fast, easy to use and ‘low-cost' molecular assay employing HLA-B*57:01 as a predictive pharmacogenomic biomarker.

In fact, a simple buccal swab is necessary to directly type patients for the presence of the HLA-B*57 region, discriminating >90% of the individuals in the majority of populations, which are HLA-B*57-negative and is eligible for a therapy containing ABC. The remaining ~7% of HLA-B*57-positive patients undergoes the second phase of nested PCR in order to be classified as HLA-B*57:01-positive (not eligible for ABC therapy) or negative.

**Figure 4:**
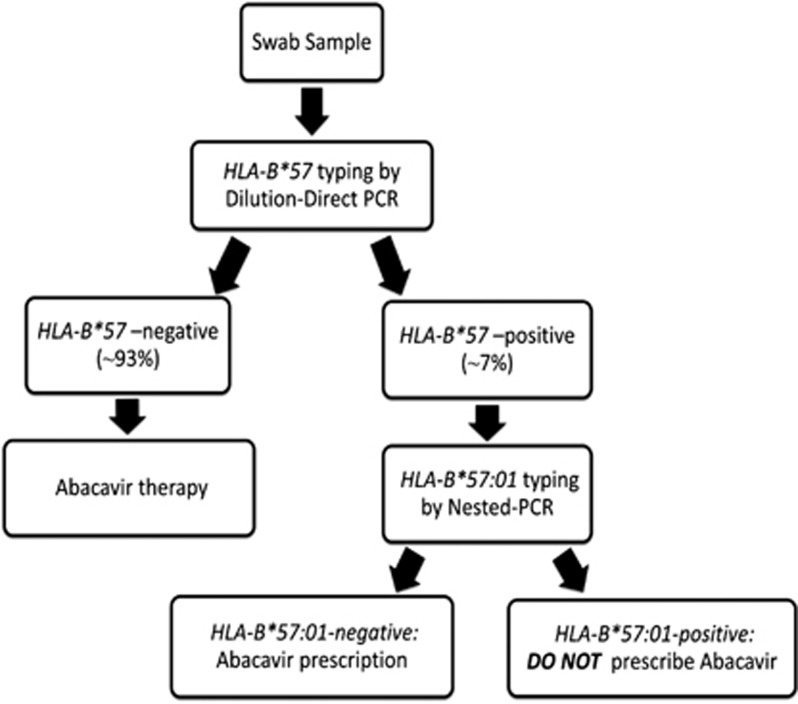
The dilution-direct/nested-PCR workflow system.

The developed dilution-direct/nested-PCR molecular assay is a robust and reliable approach for HLA-B*57:01 testing, presenting a number of advantages over current methods. First of all it is fast and simple, as it can be performed directly on the biological sample without preliminary DNA extraction and sequencing. Second, it can be performed on less infective biological samples, such as the buccal swabs. Third, the advantage of a molecular assay divided in two analytical phases allows the discrimination of >90% of patients in 90 min (dilution-direct PCR), while the remaining 7% is subjected to further 90-min analysis (nested PCR). This kind of ‘workflow' (summarized in Figure 4) and the possibility of avoiding DNA extraction and sequencing procedures result in a considerable reduction in terms of time and costs necessary for the whole genetic analysis.

The required low-cost technology for this molecular assay, consents thereby the exportation of the pharmacogenetic test in those countries and laboratories that do not dispose of the advanced technologies employed for HLA-B*57:01 typing by traditional methods.

The developed dilution-direct/nested-PCR approach supports the implementation of the HLA-B*57:01 pharmacogenetic test in order to promote the use of an efficient antiretroviral drug as ABC, even in low- and middle-income countries, where currently available approaches are often not available or not affordable.

Finally, the dilution-direct/nested PCR represents an innovative example of translational medicine as well as the application of pharmacogenetics in the clinical practice encouraging personalized, safer and cost-effective therapy.

## Figures and Tables

**Table 1:** Results of the sequenced direct/nested-PCR products

Subjects typed for HLA-B*57	*Direct/nested PCR results*	*Sequence*
Subject 1	HLA-B*57-positive/HLA-B*57:01-**positive**	HLA-B*57:01
Subject 2	HLA-B*57-positive/HLA-B*57:01-**positive**	HLA-B*57:01
Subject 3	HLA-B*57-positive/HLA-B*57:01-**positive**	HLA-B*57:01
Subject 4	HLA-B*57-positive/HLA-B*57:01-*negative*	HLA-B*57:02

The 'bold' characters are used to highlight and relate the results of Directs/Nested PCR and sequence, which are compared in the table 1. The 'italic and underline' characters instead have been used to distinguish the results obtained and confirmed in the 'subject 4' by both methods.

## References

[bib1] Crews KR Hicks JK Pui C-H Relling MV Evans WE Pharmacogenomics and individualized medicine: translating science into practice Clin Pharmacol Ther 2012 92 467 475 22948889 10.1038/clpt.2012.120 PMC3589526

[bib2] Hood L Systems biology and p4 medicine: past, present, and future Rambam Maimonides Med J 2013 4 e0012 23908862 10.5041/RMMJ.10112 PMC3678833

[bib3] Mallal S Philips E Introduction of pharmacogenetic screening to HIV clinical practice: potential benefits and challenges HIV AIDS 2007 2 13 18

[bib4] Cohen JP Overcoming regulatory and economic challenges facing pharmacogenomics N Biotechnol 2012 29 751 756 22370122 10.1016/j.nbt.2012.02.001

[bib5] Ritchie MD The success of pharmacogenomics in moving genetic association studies from bench to bedside: study design and implementation of precision medicine in the post-GWAS era Hum Genet 2012 131 1615 1626 22923055 10.1007/s00439-012-1221-z PMC3432217

[bib6] Ma SQ Lu AYH Pharmacogenetics, pharmacogenomics, and individualized medicine Pharmacol Rev 2011 63 437 459 21436344 10.1124/pr.110.003533

[bib7] Hetherington S Hughes AR Mosteller M Shortino D Baker KD Spreen W Genetic variations in HLA-B region and hypersensitivity reactions to abacavir Lancet 2002 358 1121 1122 11943262 10.1016/S0140-6736(02)08158-8

[bib8] Mallal S Nolan D Witt C Masel G Martin AM Moore C Association between presence of HLA-B*5701, HLA-DR7, HLA-DQ3 and hypersensitivity to HIV-1 reverse-transcriptase inhibitor abacavir Lancet 2002 359 727 732 11888582 10.1016/s0140-6736(02)07873-x

[bib9] Hewitt RG Abacavir hypersensitivity reaction HIV AIDS 2002 34 1137 1142 10.1086/339751 11915004

[bib10] Hetherington S McGuirk S Powell G Cutrell A Naderer O Spreen B Hypersensitivity reactions during therapy with the nucleoside reverse transcriptase inhibitor abacavir Clin Ther 2001 23 1603 1612 11726000 10.1016/s0149-2918(01)80132-6

[bib11] Mallal S Philips E Carosi G Molina J-M Warkman C Tomažič J *HLA-B*5701* screening for hypersensitivity to abacavir N Engl J Med 2008 358 568 579 18256392 10.1056/NEJMoa0706135

[bib12] Saag M Balu R Philips E Brachman P Martorell C Burman W High sensitivity of human leukocyte antigen-B*5701 as a marker for immunologically confirmed abacavir hypersensitivity in white and black patients HIV AIDS 2008 46 1111 1118 10.1086/529382 18444831

[bib13] Philips E Mallal S Successful translation of pharmacogenetics into the clinic: the abacavir example Mol Diagn Ther 2009 13 1 9 19351209 10.1007/BF03256308

[bib14] Nolan D *HLA-B*5701* screening prior to abacavir prescription: clinical and laboratory aspects Crit Rev Clin Lab Sci 2009 46 153 165 19514905 10.1080/10408360902937817

[bib15] Guo YL Shi LM Hong HX Su ZQ Fuscoe J Ning BT Studies on abacavir-induced hypersensitivity reaction: a successful example of translation of pharmacogenetics to personalized medicine Sci China Life Sci 2013 56 119 124 23393027 10.1007/s11427-013-4438-8

[bib16] Martin MA Klein TE Dong BJ Pirmohamed M Haas DW Kroetz DL Clinical pharmacogenetics implementation consortium guidelines for HLA-B genotype and abacavir dosing Clin Pharmacol Ther 2012 91 734 738 22378157 10.1038/clpt.2011.355 PMC3374459

[bib17] Stocchi L Cascella R Zampatti S Pirazzoli A Novelli G Giardina E The pharmacogenetic HLA biomarker associated to adverse abacavir reactions: comparative analysis of different genotyping methods Curr Genomics 2012 13 314 320 23204921 10.2174/138920212800793311 PMC3394119

[bib18] Giardina E Stocchi L Foti Cuzzola V Zampatti S Gambardella S Patrizi MP A fluorescence-based sequence-specific primer PCR for the screening of *HLA-B*57:01* Electrophoresis 2010 31 3525 3530 20925049 10.1002/elps.201000283

[bib19] DeJesus E Herrera G Teofilo E Gerstoft J Buendia CB Brand JD Abacavir versus zidovudine combined with lamivudine and efavirenz, for the treatment of antiretroviral-naïve HIV-infected adults Clin Infect Dis 2004 39 1038 1046 15472858 10.1086/424009

[bib20] Lucas A Nolan D Mallal S HLA-B*5701 screening for susceptibility to abacavir hypersensitivity J Antimicrob Chemother 2007 59 591 593 17317695 10.1093/jac/dkl557

[bib21] WHO (World Health Organization) Antiretroviral medicines in low- and middle-income countries: forecasts of global and regional demand for 2012–2015 AIDS Medicines and diagnostics service; HIV/AIDS Programme 2013

[bib22] WHO (World Health Organization)WHO Survey on ARV and Diagnostic Use 2012: Preliminary Results; http://www.who.int/hiv/amds/1-PP7.pdf .

[bib23] Masimirembwa C Hasler JA Pharmacogenetics in Africa, an opportunity for appropriate drug dosage regimens: in the road to personalized healthcare CPT Pharmacometrics Syst Pharmacol 2013 2 e45

